# Symbiotic Bacteria Modulate *Lymantria dispar* Immunity by Altering Community Proportions after Infection with LdMNPV

**DOI:** 10.3390/ijms24119694

**Published:** 2023-06-02

**Authors:** Peixu Zhao, Christopher Rensing, Dun Wang

**Affiliations:** 1 State Key Laboratory of Crop Stress Biology for Arid Areas, College of Plant Protection, Northwest A&F University, Yangling 712100, China; 15890058355@163.com; 2 Institute of Environmental Microbiology, College of Resource and Environment, Fujian Agriculture & Forestry University, Fuzhou 350002, China; crensing94@gmail.com

**Keywords:** symbiotic bacteria, *Lymantria dispar*, immunity, LdMNPV, infection

## Abstract

The symbiotic bacteria–insect interaction is considered to be associated with immunity and drug resistance. However, the wide variety of insect species and habitats is thought to have a significant impact on the symbiotic community, leading to disparate results. Here, we demonstrated that symbiotic bacteria regulated the immune response by changing the proportion of the Gram-positive and the Gram-negative bacterial community in *Lymantria dispar* (*L. dispar*) after infection with its viral pathogen, *L. dispar* Nucleopolyhedrovirus (LdMNPV). After oral infection, the immune deficiency pathway was activated immediately, and the expression of *Relish* was up-regulated to promote the secretion of antimicrobial peptides. Meanwhile, the abundance of the Gram-negative bacterial community increased at the same time. Moreover, the Toll pathway was not regulated in the same way as the Imd pathway was after infection. However, the change in the Toll pathway’s expression remained positively correlated to the abundance of Gram-positive bacteria. This finding implied that the ratio of Gram-negative to Gram-positive bacteria in the LdMNPV infected larvae had an effect on the immune response. Our findings revealed that the immune regulation of *L. dispar* was regulated by the relative abundance of its symbiotic bacteria at different infection times with LdMNPV, which provides a new way to understand symbiotic bacteria–insect interactions.

## 1. Introduction

Baculoviruses are a special family of viruses widely found in arthropods, including more than 600 known viruses [1]. One of their main hosts, the Lepidoptera pest, has been widely controlled by baculovirus-based biological insecticides [2,3,4]. *Lymantria dispar* (*L. dispar*) Nucleopolyhedrovirus (LdMNPV) specifically infects *L. dispar* larvae by intaking food into their midgut [5]. The occlusion bodies (OBs) of LdMNPV are dissolved under alkaline midgut (pH 10–11) conditions and proteases to release occlusion-derived virions (ODVs) [5,6]. The ODVs pass through the peritrophic membrane (PM) and infect midgut epithelium cells [7] which produce a budded virus (BV) phenotype to infect other tissues through the tracheal system [8,9,10]. Eventually, infected caterpillars climb higher branches to liquefy upside down [11,12].

Like with other pathogens, a baculovirus infection activates the immune system to provide better protection for the host [13,14,15]. Innate immunity is the only immune response in invertebrates that consists of both cellular and humoral immunity [16]. Cellular immunity is mainly provided by plasma cells and depends on phagocytosis and the inclusion of blood cells to recognize foreign bodies [16,17,18,19,20]. The humoral immune response includes antimicrobial peptides (AMPs) derived from the Toll and immune deficiency (Imd) pathways and peptides derived from prophenoloxidase and other enzymes participating in coagulation and melanization [21]. Bacteria and fungi have been shown to produce antimicrobial peptides through the expression of the Toll and Imd pathways [22]. Gram-negative bacteria mainly activated the Imd pathway, while Gram-positive bacteria and fungi mainly activated the Toll pathway [22]. The study of the antiviral response showed that melanogenesis made it possible to resist *Autographa californica* multiple nucleopolyhedrovirus (AcMNPV) infection in *Helicoverpa zea*. The Toll and Imd pathways, the JAK/STAT pathway and the MAPK pathway were thought to be activated when *Bombyx mori* was infected by *Bombyx mori* nucleopolyhedrovirus (BmNPV) [23,24,25], and RNA interference (RNAi) is also considered to be a major antiviral pathway for insects [26,27,28,29]. However, the antiviral response is thought not to be the same as the antibacterial response [30]. In *Aedes aegypti*, the Imd pathway was shown to be induced by cricket paralysis virus (CrPV) but the Toll pathway had no such a response [31]. The humoral or cellular immune response of bees was not induced by the acute bee paralysis virus (ABPV) [32]. Thus, different types of pathogens cause different immune system responses in insects.

Symbiotic bacteria are micro-organisms living in a symbiotic relationship with the host and have no effect on or even benefit the host [33,34,35]. Insect symbiotic bacteria can be divided into four types according to the degree of stability of the symbiotic bacteria–host association [36]. As the first type, obligate symbionts have been considered endocellular specialized organs in the insect body that are transmitted vertically between host generations [37]. The second type, known as facultative symbionts, have been found intracellularly or extracellularly in the hemolymph and midgut tissue [38]. Extracellular symbionts are the third type that display strong environmental plasticity and various transmission pathways [39,40]. Insect gut microbes are important extracellular symbionts that have been widely studied due to their unique location and diversity of impacts on hosts [41,42,43,44]. Finally, the fourth type are called “external symbionts,” living outside of the host’s body on a food source such as *Leucoagaricus gongylophorus* which helps leafcutter ants absorb nutrients or assimilate nitrogen [45,46]. The fourth type of symbiotic bacteria have commonly been found in insects that feed on fungi [47,48]. In the entomo-symbiotic system, the insect host is protected by producing antibacterial substances [49,50,51,52,53], regulating the expression of host immune-related genes [54,55,56], participating in immune priming and interspecies competition [57,58,59,60]. However, the results of many studies of symbiotic bacteria were mainly focused on the defense against pathogenic bacteria and fungi, and there were fewer studies on defense against viruses.

Studies have shown that symbiotic bacteria are able to affect insect communities [61] and provide essential compounds for insects, such as supplemental nitrogen for termites or complementary amino acids for *Aphidoidea* [62,63,64]. Symbiotic bacteria were also shown to participate in the metabolic activities of the host, such as hemolytic enzymes being secreted by *Serratia* and *Enterobacter*, which were beneficial to *Aedes aegypti* in terms of promoting the digestion of blood after bloodsucking [65]. In addition, insect symbiotic bacteria are involved in the immune system and in drug resistance [66,67,68,69]. Bacteria-free *Drosophila melanogaster* populations were shown to be more sensitive to oral infections by *Pseudomonas aeruginosa*, *Serratia marcescens*, and *Candida albicans*, but these opportunistic pathogens were defeated by the presence of elevated populations of *Lactobacillus plantarum* [70,71]. Intestinal micro-organisms were able to mediate responses in host immune genes, including the up-regulation of cecropin and peptidoglycan recognition proteins, thereby improving mosquito resistance to malaria parasites [72]. There is clearly a special relationship between symbiotic bacteria and the immune system. We hypothesized that changes in the symbiotic bacterial community would influence the immune system in *L. dispar* after LdMNPV infection. In this study, we selected three timepoints, 12, 24, and 72 h, of third instar larvae to investigate the changes in the symbiotic bacterial community and its effects on immune pathways.

## 2. Results

### 2.1. Effect of LdMNPV Solution with Different Concentrations on Mortality

**Figure 1:**
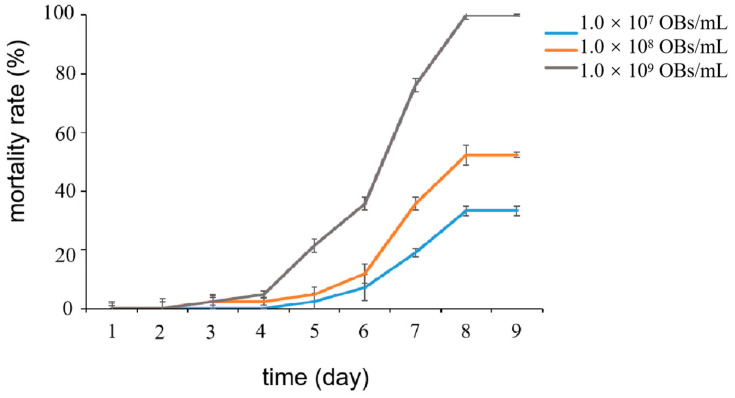
Mortality of *Lymantria dispar* (*L. dispar*) larvae after feeding LdMNPV at different concentrations.

We tested the effects of different concentrations of the LdMNPV solution on the mortality of *L. dispar* larvae. The results showed that the mortality rate of *L. dispar* larvae was able to reach 100% by feeding 1 μL of LdMNPV with a concentration of 10^9^ OBs/mL (Figure 1). Therefore, LdMNPV with a concentration of 10^9^ OBs/mL was selected for subsequent experiments.

### 2.2. Sequencing Information

**Figure 2:**
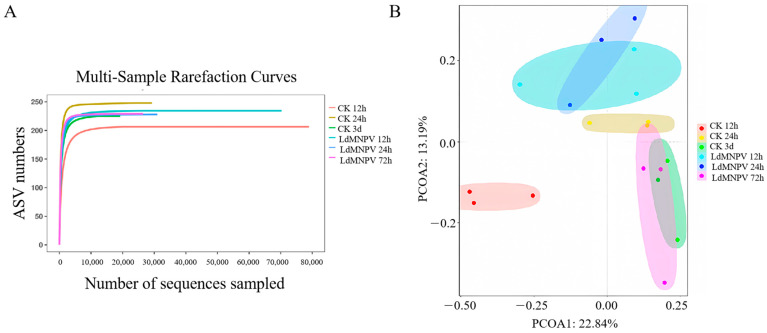
16S rDNA sequencing of symbiotic bacteria based on level of amplicon sequence variants (ASVs) of *L. dispar* larvae after viral infection. (**A**) Rarefaction curve. (**B**) PCoA plot with Bray–Curtis distance at ASV level.

A total of 1838,503 reads were obtained from 18 samples after the 16S rRNA gene (V3–V4 region) sequencing of symbiotic bacteria. After quality filtering and the removal of redundant sequences, 1,454,441 high-quality sequences were assembled into the 16S rDNA sequencing database. These high-quality sequences were clustered into 765 amplicon sequence variants (ASVs), 331 species, 278 genera, 156 families, 83 orders, 28 classes, and 19 phyla. The quantity of samples was proven to be sufficient by the rarefaction curve (Figure 2A).

### 2.3. Composition and Structure of Microbial Community of Symbiotic Bacteria

According to the principal coordinate analysis (PCoA) of symbiotic bacteria, it could be seen that the LdMNPV groups which were fed LdMNPV at a concentration of 10^7^/larva and control groups (CK) which were fed the same volume of double-distilled water showed an independent confidence ellipse at 12 and 24 h, but the difference decreased gradually with time. At 72 h, the two groups of independent confidence ellipses overlapped, indicating that the structural difference in symbiotic bacteria was not significant (Figure 2B).

**Figure 3:**
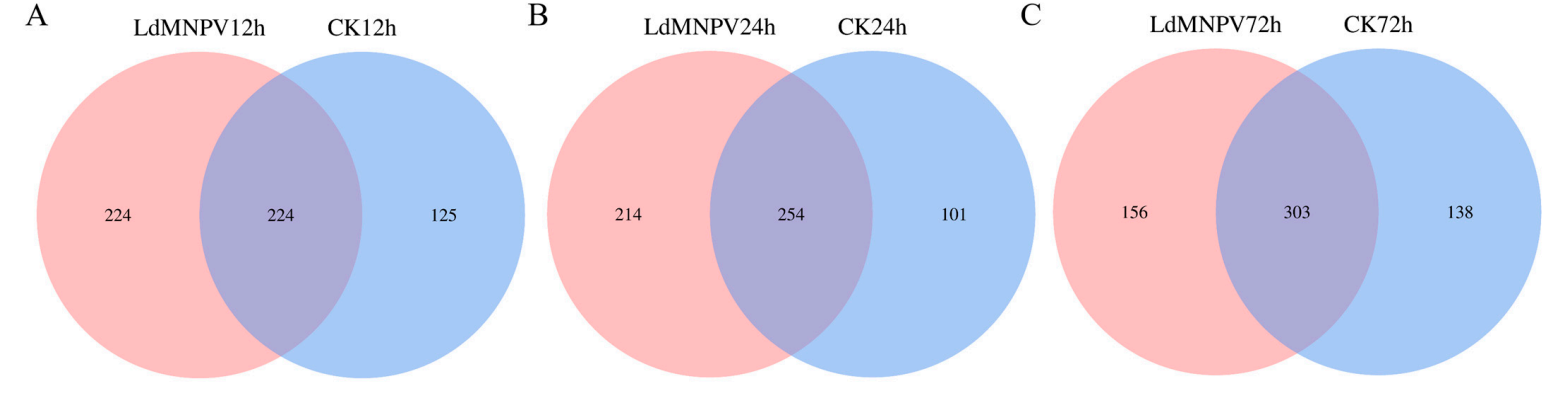
Venn diagram analysis at the level of amplicon sequence variants. Symbiotic bacteria at 12 h (**A**), 24 h (**B**) and 72 h (**C**) after *L. dispar* larvae infected by LdMNPV.

Venn diagrams showed that 224 and 125 bacterial ASVs were specifically detected in the LdMNPV and the CK groups at 12 h, and the remaining 224 ASVs overlapped (Figure 3A). At 24 h, 214 and 101 bacterial ASVs were specifically detected in the LdMNPV and CK groups, respectively, and there were 254 common ASVs (Figure 3B). There were 156 and 138 unique ASVs present in the LdMNPV and CK groups at 72 h, and there were 303 ASVs in common (Figure 3C). The principal coordinate analysis (PCoA) visualization of symbiotic bacteria demonstrated the complete separate confidence ellipses at 12 h and 24 h (Figure 2B). These results suggest that LdMNPV exposure significantly altered the ASV composition of symbiotic bacteria in *L. dispar* larvae at 12 and 24 h, but the change decreased with an increase in the infection time at 72 h.

**Figure 4:**
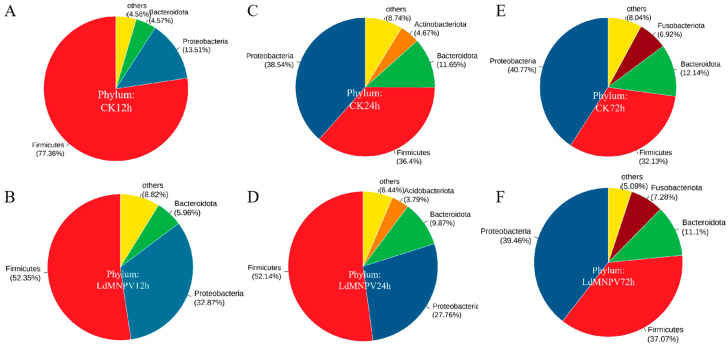
The visualization analysis of community composition using a pie chart at the phylum level. LdMNPV: LdMNPV-exposed larvae; CK: control groups. (**A**) CK groups at 12 h. (**B**) LdMNPV groups at 12 h. (**C**) CK groups at 24 h. (**D**) LdMNPV groups at 24 h. (**E**) CK groups at 72 h. (**F**) LdMNPV groups at 72.

The analysis at the phylum level showed that Firmicutes and Proteobacteria were the dominant bacterial phyla in the symbiotic community (Figure 4A–F). The data suggested that LdMNPV infection increased Proteobacterial abundance and reduced Firmicute abundance at 12 h (Figure 4A,B). Firmicutes were more abundant and Proteobacteria were less abundant in the LdMNPV groups after 24 h (Figure 4C,D). The relative abundance of symbiotic bacteria did not differ much at the phylum level after 72 h (Figure 4E,F). These results indicated that at the phylum level the relative abundance of symbiotic bacteria was significantly different from that of the CK and LdMNPV groups at 12 and 24 h, and that there were few differences at 72 h between the two groups. It was notable that the change in the relative abundance of symbiotic bacteria was mainly different at a ration of Firmicutes (Gram-positive bacteria) and Proteobacteria (Gram-negative bacteria).

**Figure 5:**
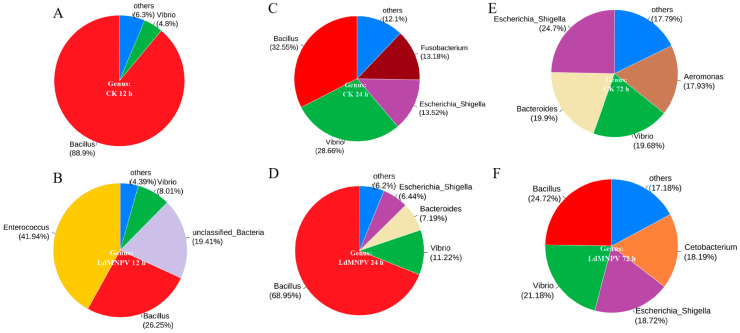
The visualization analysis of community composition using a pie chart at the genus level. LdMNPV: LdMNPV-exposed larvae; CK: control groups. (**A**) CK groups at 12 h. (**B**) LdMNPV groups at 12 h. (**C**) CK groups at 24 h. (**D**) LdMNPV groups at 24 h. (**E**) CK groups at 72 h. (**F**) LdMNPV groups at 72 h.

Furthermore, at the genus level, we selected several symbiotic bacteria present at a higher percentage for community relative abundance analysis. The results showed that the abundance of Enterococcus and unclassified bacteria increased by 41.94% and 19.41% at 12 h in the LdMNPV groups. Meanwhile, Bacillus (26.25%) was reduced by 62.75% in the LdMNPV groups compared to the CK groups at 12 h (Figure 5A,B). At 24 h, the Bacillus (68.95%) community proportion was doubled and the Vibrio (11.22%) community proportion halved in the LdMNPV groups compared to those in the CK groups (Figure 5C,D). Bacillus (24.72%) was still present in the LdMNPV groups but not present in the CK groups at 72 h (Figure 5E,F). Thus, at the genus level, LdMNPV infection mainly affected the abundance of Bacillus and Vibrio in the symbiotic bacterial community, except for significantly changing Enterococcus and unclassified bacteria abundance at 12 h.

### 2.4. Host Immunity-Related Genes Differentially Expressed following LdMNPV Infection

**Figure 6:**
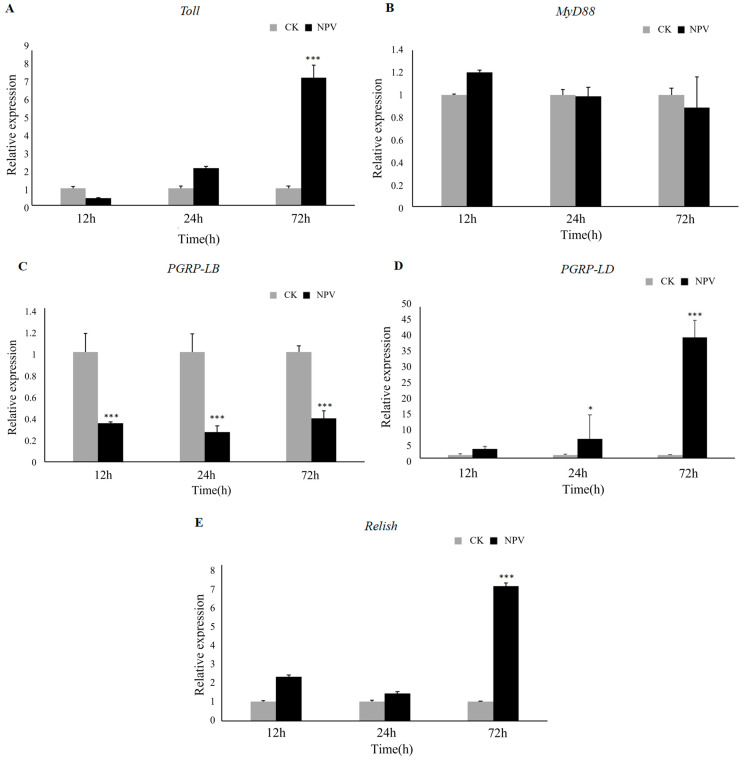
Expression analysis of the Toll and Imd pathway’s key genes of *L. dispar* larvae after infection by LdMNPV. (**A**,**B**) The *Toll* and *MyD88* gene expression at 12, 24 and 72 h. (**C**,**D**) The *PGRP-LB* and *PGRP-LD* expression at 12, 24 and 72 h. (**E**) *Relish* gene expression at 12, 24 and 72 h. Each value represents the mean (±SE) of three replications. Different letters in the bar graph indicate significant differences (*p* < 0.05). Additionally, “*” means *p* < 0.05 and “***” means *p* < 0.001.

The expression of immune genes, including those of the Toll pathway, the Imd pathway and the NF-κβ pathway, was analyzed in *L. dispar* larvae after LdMNPV infection. The results showed that in the Toll pathway, *Toll* gene expression was down-regulated at 12 h, up-regulated at 24 h and showed seven-fold up-regulation at 72 h in the LdMNPV groups compared to the CK groups (Figure 6A). However, the expression of the *MyD88* gene, a key gene of the Toll pathway, was not significantly different after LdMNPV infection (Figure 6B). The expression of the gene encoding peptidoglycan recognition protein-B (*PGRP-LB*), a repressor in the Imd pathway, was down-regulated at 12, 24, and 72 h (Figure 6C). Moreover, the expression of the gene encoding peptidoglycan recognition protein-D (*PGRP-LD*), an activator in the Imd pathway, was up-regulated at 12 h, showed 7-fold up-regulation at 24 h, and was up-regulated 40-fold at 72 h (Figure 6D). In addition, the expression of the *Relish* gene, a key activator of the NF-κβ pathway, showed up-regulation at 12 and 24 h, and seven-fold up-regulation at 72 h in the LdMNPV groups (Figure 6E). These results suggested that the *L. dispar* immune system was not fully activated after LdMNPV infection. The Toll pathway did not seem to be induced and the *Toll* gene even showed down-regulation at 12 h after LdMNPV infection. Moreover, the expression of the Imd pathway was activated after LdMNPV infection as was the NF-κβ pathway, which is a key signal pathway regulating the expression and generation of AMPs.

## 3. Discussion

Micro-organisms are widely distributed in nature, and many of them have formed complex and diverse relationships with insects in the course of evolution [32,61]. Symbiotic relationships, as one of them, played an important role in the growth and development of insects and influenced the establishment of an ecological niche for insects [45,73,74,75,76]. Symbiotic bacteria have been shown to participate in the physiological and biochemical processes of insects; particularly, the effects of symbiotic bacteria on insect detoxification and the immune system have been given more attention [57,77]. To further understand the effect of symbiotic bacteria in the immunization against viruses, this study analyzed the variations of symbiotic bacteria in *L. dispar* larvae after LdMNPV infection and the relationship between these changes and host immune signaling pathways.

Our results indicated that an LdMNPV infection has significant effects on the symbiotic bacterial community in *L. dispar* larvae. The number of ASVs in LdMNPV groups was significantly higher than that in CK groups at 12, and 24 h, and was similar between the two groups at 72 h. This implied that an LdMNPV infection led to differences in symbiotic bacterial species and increased the species of symbiotic bacteria at 12 and 24 h after infection. This was the similar to the changes in manganese ion, aconitine, and nicotine stress in *L. dispar* and *Spodoptera exigua* nuclear polyhedrosis virus (SeMNPV) infection in *Spodoptera exigua* [78,79]. However, the amount of gut microbiota species was decreased after baculovirus infection in *Helicoverpa armigera* and bidensovirus (BDV) infection in *Bombyx mori* [80,81]. These different trends in microbiota changes between these studies were probably caused by different sample times or different sample species. The community abundance pie chart at the phylum level also showed the changes in symbiotic bacteria in *L. dispar* larvae infected with LdMNPV. Firmicutes and Proteobacteria were the dominant phyla of the symbiotic bacterial community in *L. dispar* larvae, which was same as what has been observed in previous studies [42,79]. Similarly to the results of the studies on avermectin, aconitine and nicotine stress, Firmicutes as Gram-positive bacteria were increased and Proteobacteria as Gram-negative bacteria were decreased compared to CK groups at 24 and 72 h [79,82]. In this study, we found that the community relative abundance of Firmicutes was decreased by 25% and that of Proteobacteria was increased by nearly 20% at 12 h in LdMNPV groups compared to CK groups. The changes in the symbiotic bacterial community were the opposite of those observed at 24 and 72 h. This indicated that there was a significant difference in the changes in the symbiotic bacterial community between the initial period and final period after LdMNPV infection. This difference was probably caused by the different stages of virus infection [83].

Furthermore, the abundance at the genus level changed dramatically, indicating that LdMNPV had changed the balance of symbiotic bacteria. Bacillae as Gram-positive bacteria have been used as pesticide in insects, the most representative being *Bacillus thuringiensis* (Bt) [84]. However, many studies showed that Bacillus was also able to help isolate antibacterial, anti-inflammatory and antiviral compounds [85,86,87]. In this study, the relative abundance of Bacillus was dominant in the CK groups at both 12 h and 24 h, indicating that Bacillus would not harm the host. In the LdMNPV groups, the relative abundance of Bacillus was dominant at 12, 24 and 72 h. The results showed that Bacillus in *L. dispar* larvae increased in relative abundance when the latter were infected with baculovirus. This increased relative abundance probably helped the host defend against viral infection and reduce inflammation. Enterococcus, one of the predominant members of the gut microbial community, has been found to play a crucial role in metabolic adaptability against pathogenic or plant toxins and anti-herbivore defense, including that against *B. mori*, *Helicoverpa zea*, and *Porthetria dispar* [88,89]. *Enterococcus faecalis* was found in *L. dispar* and was able to acidify its local environment to defend against pathogenic toxins that were activated under alkaline conditions, such as *Bacillus thuringiensis* [90]. Interestingly, LdMNPV was one of the pathogens that was dissolved under alkaline conditions [5,6]. It was rationally explained that the Enterococcus community increased by 41.94% at 12 h in LdMNPV groups. Vibrio as a Gram-negative bacterium only displayed a significant difference at 24 h in LdMNPV groups compared to that in CK groups. This result was possibly due to the optimal conditions for Vibrio being alkaline, and the increased relative abundance of the Bacillus ratio resulted in an acidic environment, which was not conducive to the reproduction of Vibrio [91]. In addition, due to the destruction of insect immunity and normal symbiotic homeostasis by virus infection, the LdMNPV groups showed an increase in unclassified bacteria at 12 h. These results suggested that LdMNPV infection was able to alter the normal symbiotic bacterial community of *L. dispar* larvae, and the changes in the abundance of these symbiotic bacteria were closely related to the function of the immune system.

The Toll and Imd pathway were shown to respond to infection by different viruses in the insect immunity system [92,93]. They produced AMPs by activating the NF-κB pathway which has been shown to possess antiviral activity [94,95]. The Toll pathway was not as rapidly responsive to LdMNPV infection in this study, and expression even decreased at the early stage of infection. This was similar to the finding of Alphaviruses infecting *D. melanogaster* and *Spodoptera frugiperda* being infected with ascovirus [96,97]. In the Imd pathway, *PGRP-LB* as a suppressor gene, showed significantly decreased expression over time. *PGRP-LD*, as a positive regulatory gene, displayed high levels of expression at all time points after LdMNPV infection. The *Relish* gene that activated the NF-κB pathway to produce AMPs showed similar results to those of the Imd pathway. These results indicated that the Imd pathway was activated rapidly after LdMNPV infection in *L. dispar* larvae, while the Toll pathway was delayed. Meanwhile, the Toll and Imd pathways were thought to be activated by Gram-positive and Gram-negative bacteria [22]. The connection between the change in the composition of the symbiotic bacterial community and the expression of immune genes was clearly visible. At the phylum level, the decrease in the abundance of Gram-positive bacteria had an effect on the decrease in *Toll* gene expression at 12 h after infection, while the increase in the Gram-negative bacterial community corresponded with an increase in the expression of genes encoding the Imd pathway. Similarly, *Toll* gene expression was increased with the increase in Gram-positive bacteria at 24 h after infection, but the decrease in Gram-negative bacteria did not affect the increase in the expression of the Imd pathway. This is probably correlated to the continuous inhibition of *PGRP-LB*. *PGRP-LB* is believed to lyse peptidoglycan secreted by symbiotic bacteria at a low level, which could inhibit the overresponse of the Imd pathway and protect the symbiotic bacteria [98]. The abundance of Bacillus (Gram-positive bacteria) increased, which was synchronous with the increase in *Toll* gene expression at 24 h. These results suggested that the ratio of Gram-negative to Gram-positive bacteria in the symbiotic bacterial community had an effect on the immune response after LdMNPV infection.

In summary, LdMNPV infection significantly changed the symbiotic bacteria community structure and composition. These changes probably helped the host to resist the virus. Moreover, the response of the Toll and Imd pathway after the LdMNPV infection of *L. dispar* larvae was correlated with changes in the symbiotic bacterial community. The increased relative abundance of Gram-negative bacteria in the symbiotic community could activate the Imd pathway to up-regulate the expression of the NF-κB pathway to produce AMPs. The increased relative abundance of Gram-positive bacteria in the symbiotic community was only able to up-regulate the expression of the *Toll* gene but was shown to affect the Toll pathway less. Moreover, the regulation changed with the alteration in the symbiotic bacterial community at different infection times. Like the fact that *Tenebrio molitor* was able to quickly kill 99.5% of *Staphylococcus aureus* within 30 min to 3 h [99], the insect immune system displayed different working states after infection with different pathogens. These findings improved our understanding of the immune system’s relationship with the symbiotic bacterial community after viral infection and provide a new way to better understand the symbiotic bacteria–insect interaction.

## 4. Materials and Methods

### 4.1. Insect and Virus

*L. dispar* larvae were grown in the laboratory as previously described [100]. LdMNPV was prepared as described previously, with slight modifications [100]. LdMNPV-infected larvae were homogenized with 1× phosphate-buffered saline (PBS), filtered through multi-layer gauze, and then centrifuged at 6000 rpm at 4 °C for 30 min. The sediment was then stored at 4 °C for further processing. The collected viruses were diluted to 1 × 10^9^ OBs/mL and stored at −20 °C.

### 4.2. Bioassays

*L. dispar* larvae at the early third instar were fed 1 μL of the LdMNPV solution at concentrations of 10^7^, 10^8^ and 10^9^ OBs/mL after starvation treatment. Mortality was recorded every 12 h until the death of all of the larvae. Thirty larvae were used for each treatment, and the experiment was performed three times. Mortality was expressed as the percentage of dead larvae using the Kaplan–Meier method (GraphPad Prism version 5).

### 4.3. Sample Preparation

The third-instar larvae were randomly divided into LdMNPV groups and control groups. The LdMNPV groups were fed 1 μL of the LdMNPV solution at a concentration of 1 × 10^9^ OBs/mL. The CK groups were fed the same volume of double-distilled water. The treated larvae were placed into an insect rearing box with the same artificial diet. Two live larvae were chosen as samples at 12, 24, and 72 h, and each treatment group was independently repeated three times. The samples were treated with 75% alcohol to be disinfected and snap frozen in liquid nitrogen. Then, the samples were stored at −80 °C for symbiotic sequencing.

### 4.4. DNA Extraction, PCR Amplification and Miseq Sequencing

The DNA of the whole larva was extracted using TIANamp Stool DNA Kit (TIANGEN, Beijing, China) according to the manufacturer’s instructions. DNA quality was checked using a NanoDrop 2000 UV-vis spectrophotometer (Thermo Fisher Scientific, Wilmington, DE, USA) and 2% agarose gels. The V3–V4 region of the symbiotic microbial 16S rRNA gene was amplified using gene-specific primers 338F (5′-ACTCCTACGGGAGGCAGCAG-3′) and 806R (5′-GGACTACHVGGGTWTCTAAT-3′) [101]. Amplifications were carried out with Q5 High-Fidelity DNA Polymerase (NEB, Ipswich, MA, USA) according to the manufacturer’s instructions. Amplicons were purified using VAHTSTM DNA Clean Beads (Vazyme, Nanjing, China) and quantified using Quant-iT™ dsDNA HS Reagent (Thermo Fisher Scientific, Wilmington, DE, USA) according to the manufacturers’ protocols. Each purified product was sequenced using the Illumina HiSeq platform (Biomarker Technologies, Beijing, China).

### 4.5. 16S rRNA Gene Sequence Analysis

The Isanger Cloud platform (https://www.i-sanger.com/ accessed on 20 February 2023) was employed for all bioinformatics analyses. Raw sequences were demultiplexed, quality-filtered via Trimmomatic, and merged via FLASH using the following criteria: (a) the reads were truncated at any site receiving an average quality score of <20 over a 50 base pair (bp) sliding window; (b) primers were exactly matched, allowing up to two nucleotide mismatches, and reads containing ambiguous bases were removed; (c) sequences with overlaps longer than 10 bp were merged, according to their overlap sequence. Amplicon sequence variants (ASVs) were clustered with a 97% similarity cutoff using Usearch v10, and chimeric sequences were identified and removed using QIIME2 [102]. The rarefaction curve and community pies for each group were plotted using the Lianchuan Biocloud platform to determine community abundance and sequencing data [103]. Venn diagrams were created using jvenn to show unique and shared ASVs [104]. PCoA analysis was performed on the Tutools platform (https://www.cloudtutu.com/ accessed on 1 March 2023).

### 4.6. Quantitative Real-Time RT-PCR Analysis

At 12, 24, and 72 h, three larvae from each group were collected separately as one replicate. Each biological sample consisted of three replicates. Total RNA was extracted using the TRIzol reagent (Invitrogen, Carlsbad, CA, USA) following the manufacturer’s protocols. The quality of RNA samples was determined using 1% agarose gel, and the concentration was measured using a NanoDrop 2000 spectrophotometer (Thermo Fisher Scientific Inc., Waltham, MA, USA). Total RNA was treated with DNase I (TaKaRa Bio Inc., Otsu, Shiga, Japan) to remove the residual genomic DNA. Each sample was reverse-transcribed into cDNA using PrimeScript RT Reagent Kit Perfect Real Time (TaKaRa, Bio Inc., Otsu, Shiga, Japan) according to the manufacturer’s instructions. The cDNA was stored at −20 °C for further analysis. The sequence-specific primers were designed using the Primer Premier 5.0 software (PREMIER Biosoft International, Palo Alto, CA, USA). The relative expression levels of the immunity genes were determined via qRT-PCR in Rotor-Gene Q Real-Time Thermal Cycler (Qiagen, Hilden, Germany) using the commercial kit SYBR Premix Ex TaqTM II (TaKaRa Bio Inc., Otsu, Shiga, Japan). The PCR procedure was implemented as follows: 95 °C for 3 min, followed by 40 cycles of 95 °C for 15 s and of 55 °C for 30 s. A melting curve was obtained from 60 °C to 90 °C, with a 0.5 °C rise in temperature every 5 s to test the specificity of the amplified products. The expression levels of mRNAs were calculated according to the 2^−ΔΔCT^ method [105]. EF1α gene (forward primer 5’–3’: TTTGCCTTCCTTGCGCTCAACA; reverse primer 5’–3’: TGTAAAGCAGCTGATCGTGGGT) was used as the reference gene to normalize the transcription data.

### 4.7. Statistical Analysis

The experimental data were expressed as the mean ± standard error and analyzed statistically via IBM SPSS (Version 19.0, SPSS Inc., Chicago, IL, USA). For the analysis of statistical differences between two groups, Student’s *t*-tests were used. Fisher’s protected least significant difference (LSD) test was used to adjust the separation of means. The significance levels of these tests were set at *p* < 0.05.

## 5. Conclusions

After LdMNPV infection of *L. dispar* larvae, the host symbiotic bacteria displayed a remarkable change. The abundance of Gram-negative bacteria in the host increased with the high expression of the Imd pathway, while the abundance of Gram-positive bacteria decreased with the low expression of the Toll pathway. The Toll and Imd pathway, as a key immune pathway in insects, was positively correlated with changes in the proportion of the symbiotic community. In conclusion, symbiotic bacteria were able to affect the immune system of the host through changing the composition of the community after virus infection. These findings deepen our understanding of symbiotic bacteria–host interaction.

## Data Availability

All the individual data are gathered in the Supplemental Materials (Tables S1–S5).

## Supplementary Materials

The following supporting information can be downloaded at https://www.mdpi.com/article/10.3390/ijms24119694/s1.
