# Supplementary material for: Symbiotic Bacteria Modulate Lymantria dispar Immunity by Altering Community Proportions after Infection with LdMNPV
Source: Int J Mol Sci. 2023 Jun 2;24(11):9694. doi: 10.3390/ijms24119694 (PMC10254028; doi:10.3390/ijms24119694)
Supplement: Supplementary file 1 [file ijms-24-09694-s001.zip › ijms-2407228-supplementary.pdf]

Supplementary Table S1. Gene-specific primers for q-RT-PCR used in this study.

Gene Name	Forward Primer (5'-3')	Reverse Primer (5'-3')
TOLL	TCGCACATCAAGATGCTGACA	ACCGACAACCCAATCCCTAT
MyD88	GCCGCTTGTCCTATCTTGC	CATTGAACATTCTCGCCACC
PGRPB	CAAATCTGCCTCCTCAAGCC	ACCCGGACACTCGGTATCAC
PGRPD	ATGAAGGACGAGGGTGGAAG	ACCTAAGAGTTTGTAGTTGGGTTGT
Relish	CATCGCAACAAGTGCCTCA	GACATAACGAAACCGGAAATGA

Supplementary Table S2. Sequencing data quality assessments.

Sample ID	Raw Reads	Clean Reads	Denoised Reads	Merged Reads	Nonchimeric Reads
CK12h1	44,867	44,641	44,239	36,930	35,612
CK12h2	117,261	117,008	116,631	110,783	108,780
CK12h3	111,527	111,223	110,942	105,142	102,954
CK24h1	83,879	83,640	83,381	77,915	75,820
CK24h2	89,001	88,792	88,447	82,123	79,523
CK24h3	83,059	82,863	82,396	76,487	73,945
CK72h1	42,840	42,703	42,437	37,117	35,683
CK72h2	88,378	88,146	87,604	78,331	74,916
CK72h3	48,252	48,118	47,889	42,918	41,218
LdMNPV12h1	95,397	95,134	94,803	88,555	85,623
LdMNPV12h2	113,865	113,592	113,160	106,334	104,388
LdMNPV12h3	80,864	80,622	80,272	74,362	71,913
LdMNPV24h1	83,498	83,275	82,999	76,561	74,204
LdMNPV24h2	77,729	77,532	77,165	71,326	69,030
LdMNPV24h3	85,126	84,920	84,582	78,137	75,669
LdMNPV72h1	66,562	66,401	66,030	59,587	57,408
LdMNPV72h2	66,588	66,413	66,062	59,053	57,174
LdMNPV72h3	79,796	79,418	78,452	59,063	55,304

Supplementary Table S3. Statistical table of species by rank.

Sample	Kindom	Phylum	Class	Order	Family	Genus
CK12h1	1	16	24	53	85	132
CK12h2	1	16	23	52	85	121
CK12h3	1	15	21	48	78	118
CK24h1	1	17	24	57	95	150
CK24h2	1	16	25	61	100	150
CK24h3	1	15	20	50	88	139
CK72h1	1	16	22	50	86	122
CK72h2	1	16	23	58	106	168
CK72h3	1	15	23	54	87	128
LdMNPV12h1	1	17	25	56	92	136
LdMNPV12h2	1	17	24	54	92	133
LdMNPV12h3	1	15	22	52	91	140
LdMNPV24h1	1	15	21	51	87	135
LdMNPV24h2	1	16	22	57	91	138
LdMNPV24h3	1	15	22	52	90	133
LdMNPV72h1	1	16	22	53	85	134
LdMNPV72h2	1	17	24	60	96	154
Total	1	19	28	83	156	278

Supplementary Table S4. Phylum level of species abundance.

Phylum	Acidobacteriota	Actinobacteriota	Bacteroidota	Cyanobacteria	Deferribacterota	Desulfobacterota	Firmicutes	Fusobacteriota	Gemmatimonadota	Nitrospirota	Proteobacteria	Verrucomicrobiota	Unclassified_ Bacteria
CK12h1	413	582	1789	66	87	38	10257	340	18	128	4499	342	136
CK12h2	994	736	2259	90	41	163	58252	975	49	287	7466	382	272
CK12h3	535	337	1432	494	15	163	3489	134	65	201	26275	278	45634
CK24h1	1135	978	2802	134	27	325	13367	1014	122	455	8066	449	348
CK24h2	1011	893	3218	236	58	483	7664	1148	148	648	10820	571	269
CK24h3	1009	1599	2649	0	0	258	6050	1184	98	330	9784	272	372
CK72h1	660	333	1500	119	28	82	3507	511	75	323	4300	128	25
CK72h2	514	902	2333	820	37	233	7797	1959	91	472	9750	232	493
CK72h3	568	671	1675	77	29	189	3278	672	29	189	4453	204	206
LdMNPV12h1	822	1436	2445	8	71	235	8662	935	271	491	11140	761	1932
LdMNPV12h2	970	839	1649	17	36	282	38328	713	135	315	17788	224	8728
LdMNPV12h3	1227	1428	2475	72	28	142	10736	1365	230	409	7315	316	635
LdMNPV24h1	1132	1108	2893	31	134	298	12398	1076	117	269	8295	372	898
LdMNPV24h2	715	736	2458	212	0	124	5428	493	229	215	9299	521	9455
LdMNPV24h3	1007	460	2685	104	64	292	17053	993	0	436	7388	297	135
LdMNPV72h1	382	438	2146	0	30	247	6740	681	98	274	6847	358	509
LdMNPV72h2	313	718	1987	18	69	166	6490	1049	46	338	6714	310	195
LdMNPV72h3	290	663	1987	774	12	291	7209	2289	74	321	8197	302	341

Supplementary Table S5. Genus level species abundance.

Genus	Acidithiobacillus	Aeromonas	Akkermansia	Bacillus	Bacteroides	Enterobacter	Enterococcus	Escherichia_Shigella	Fusobacterium	Lactiplantibacillus	Neisseria	Pediococcus	Sphingomonas	Streptococcus	Vibrio
CK12h1	155	49	342	7079	662	249	119	915	254	245	74	115	138	227	900
CK12h2	405	93	382	53152	783	453	152	753	681	451	185	411	334	653	2349
CK12h3	171	137	278	76	604	224	83	375	44	409	223	303	406	391	1535
CK24h1	490	24	413	6335	711	518	120	413	837	541	362	555	292	694	2352
CK24h2	502	225	571	233	1066	859	337	977	968	752	341	555	367	614	2088
CK24h3	414	120	272	133	713	467	19	1392	907	593	374	303	566	1015	1459
CK72h1	238	61	128	49	477	213	168	567	308	426	175	312	243	290	528
CK72h2	282	1420	232	325	584	359	188	1112	454	432	345	443	220	548	725
CK72h3	214	134	204	87	732	224	81	546	437	269	135	345	204	346	520
LdMNPV12h1	328	135	708	325	772	617	421	749	744	1015	524	549	467	1316	2155
LdMNPV12h2	305	0	191	11823	731	457	21967	577	565	805	466	395	0	447	1350
LdMNPV12h3	300	96	271	3126	678	702	2016	458	1075	733	951	398	416	509	1153
LdMNPV24h1	279	86	372	6434	1095	108	49	925	612	621	505	469	357	615	1516
LdMNPV24h2	282	250	521	93	913	498	182	450	387	542	331	405	399	760	1430
LdMNPV24h3	251	131	297	11117	734	396	52	713	759	958	280	425	331	542	1341
LdMNPV72h1	351	359	358	1740	722	0	185	786	288	485	208	570	331	554	1066
LdMNPV72h2	346	430	310	1052	969	517	178	722	336	472	243	306	227	549	1471
LdMNPV72h3	75	1533	302	692	731	507	304	1131	340	177	142	118	449	368	448
